# Design and Experimental Investigation of a Compact Traveling-Wave Piezoelectric Angular Motion Motor

**DOI:** 10.3390/mi17091000

**Published:** 2026-08-24

**Authors:** Laurynas Šišovas, Andrius Čeponis

**Affiliations:** 1 Department of Aeronautical Engineering, Antanas Gustaitis Aviation Institute, Vilnius Gediminas Technical University, Linkmenų Str. 28-4, LT-08217 Vilnius, Lithuania; 2 Institute of Mechanical Science, Faculty of Mechanics, Vilnius Gediminas Technical University, Plytinės Str., 25, LT-10105 Vilnius, Lithuania

**Keywords:** traveling wave, angular motor, piezoelectric motor

## Abstract

This paper presents the design, numerical analysis, and experimental investigation of a compact traveling-wave piezoelectric motor for continuous and incremental angular motion. The key advancement of the proposed design is a compact coaxial direct-drive architecture that combines a single ring-shaped piezoelectric stator with four independently excited electrode sections, three discrete spherical contact elements, a cone-shaped rotor, and an adjustable spring-based preload mechanism. In contrast to conventional traveling-wave motors employing continuous annular or toothed contact interfaces, the proposed configuration localizes the stator–rotor interaction at three predefined contact points while allowing both continuous bidirectional rotation and incremental angular positioning within the same actuator. Numerical analysis identified the operating mode at 39.95 kHz and confirmed the formation of elliptical displacement trajectories at the spherical contact elements. The calculated resonance frequency and effective electromechanical coupling coefficient were 39.93 kHz and 4.36%, respectively. Experimental measurements showed resonance of 39.94 kHz with an effective coupling coefficient of 4.47%. The motor achieved a maximum rotational speed of 87 ± 2.2 RPM at 180 V_p-p_. The maximum stall torque reached approximately 8.3 N·mm and 8.4 N·mm for clockwise (CW) and counterclockwise (CCW), respectively, at 180 V_p-p_. Depending on the excitation amplitude, the angular step varied from 0.082 ± 0.015° to 3.038 ± 0.120°. The results confirm that the proposed compact motor can provide controllable continuous rotation and incremental angular positioning.

## 1. Introduction

High-precision angular motion is an essential function in modern optical, photonic, biomedical, robotic, and aerospace systems. Applications such as optical beam steering, lens and filter alignment, scanning microscopy, image stabilization, antenna orientation, and free-space optical communication require compact actuators capable of providing continuous rotary motion, fast response, high repeatability, and stable positioning. As these systems become increasingly compact, the dimensions, mass, integration complexity, and dynamic performance of the motion-generating components become important design constraints [1,2,3].

Conventional angular positioning mechanisms are commonly based on electromagnetic motors combined with gearboxes, couplings, bearings, and braking mechanisms [4,5]. Although such systems are technologically mature, their application in compact precision devices may be restricted by backlash, mechanical transmission losses, electromagnetic interference, and limited scalability [6,7]. Piezoelectric motors represent an attractive alternative because they convert electrical energy directly into mechanical motion through the inverse piezoelectric effect. Their main advantages include high torque density at low rotational speed, rapid response, high positioning resolution, self-locking capability, compact construction, and operation without an externally generated magnetic field [8,9,10]. These characteristics make piezoelectric motors particularly suitable for direct-drive positioning systems in which size, accuracy, and structural simplicity are critical.

Piezoelectric motors can be classified according to their excitation method, vibration type, and output motion. Among resonant piezoelectric drives, traveling-wave ultrasonic motors are widely used for generating continuous rotary motion [11]. Their operation is generally based on the superposition of two spatially orthogonal vibration modes having identical or closely matched resonant frequencies. When these modes are excited by harmonic signals with an appropriate temporal phase difference, a traveling deformation wave is generated along the stator. Points located on the stator contact surface follow elliptical trajectories composed of normal and tangential displacement components. The tangential component transfers motion to the rotor through friction, whereas the normal component periodically controls the mechanical contact between the stator and rotor. Reversing the phase sequence of the excitation signals changes the propagation direction of the traveling wave and, consequently, the direction of rotor rotation [12,13,14,15]. Ring-type stators are among the most frequently used structures in traveling-wave rotary piezoelectric motors because their axial symmetry is well suited for generating circumferential waves and continuous angular motion [16]. Conventional designs usually contain a metallic elastic ring, segmented piezoceramic elements, a toothed contact surface, a rotor, and a mechanical preload system. Teeth are commonly introduced to amplify tangential displacement at the contact interface. However, they increase manufacturing complexity and may introduce local stress concentration, deformation, or instability under high preload [17]. Moreover, the output characteristics of traveling-wave motors are strongly influenced by the contact geometry, friction conditions, matching of the operating vibration modes, and magnitude of the stator–rotor preload. Insufficient preload can result in unstable contact and slipping, whereas excessive preload increases mechanical damping, frictional losses, and wear [18].

The continuing miniaturization of precision positioning devices creates a need for rotary motors with simpler structures, smaller installation volumes, and contact interfaces that can be reliably controlled. The use of discrete spherical contacts is one possible approach for concentrating the interaction between the stator and rotor at predefined locations [19]. Such contacts can transfer the local elliptical motion of the stator to the rotor while providing clearly defined contact points for analysis of displacement trajectories, preload distribution, and frictional interaction. Spherical and semispherical contacts have previously been applied in piezoelectric rotary motors and multi-degree-of-freedom positioning devices. Nevertheless, their use in compact ring-type traveling-wave motors remains less extensively investigated than conventional continuous annular contact interfaces [20,21].

The main motivation of this study is to develop a compact direct-drive angular motor with a simplified design while preserving controllable continuous and incremental angular motion. Several coupled challenges arise in such a design. First, a stable circumferential bending vibration must be generated within a compact ring-shaped piezoelectric stator. Second, the micrometer-scale tangential and normal vibration components must be efficiently transmitted to the rotor through a limited number of contact points. To address these challenges, the proposed motor combines four-phase excitation of a single ring-shaped piezoelectric stator, three discrete spherical contact elements, and an adjustable spring-based preload mechanism integrated into a coaxial direct-drive structure. The key advancement of the proposed design is the integration of a single piezoelectric ring with four independently excited electrode sections and three discrete spherical contacts acting on a cone-shaped rotor. The spherical contacts localize the frictional interaction at predefined positions and reduce the sensitivity of the interface to small deviations in flatness, parallelism, and relative inclination of the contacting surfaces. The same motor configuration is used to generate both bidirectional continuous rotation and controllable incremental angular motion. The numerical investigation was performed to identify the operating vibration mode, determine the resonant characteristics of the stator, and evaluate the displacement trajectories generated at the spherical contact points. The experimental investigation was subsequently carried out to validate the operating principle and evaluate the electrical and mechanical characteristics of the fabricated motor prototype.

## 2. Motor Design and Operation Principle

The proposed piezoelectric motor was designed as a compact coaxial actuator capable of generating continuous bidirectional angular motion. The motor employs a traveling-wave operating principle, in which the vibration generated by a ring-shaped piezoelectric stator is transferred to a cone-shaped rotor through three discrete spherical contact elements. The piezoelectric stator, stainless steel rotor, stainless steel output shaft, bearing support, and adjustable preload mechanism are integrated into a cylindrical motor body. The surface roughness of the rotor contact region was not quantitatively characterized for the present proof-of-concept prototype. Therefore, no specific roughness parameter is used in this study.

**Figure 1:**
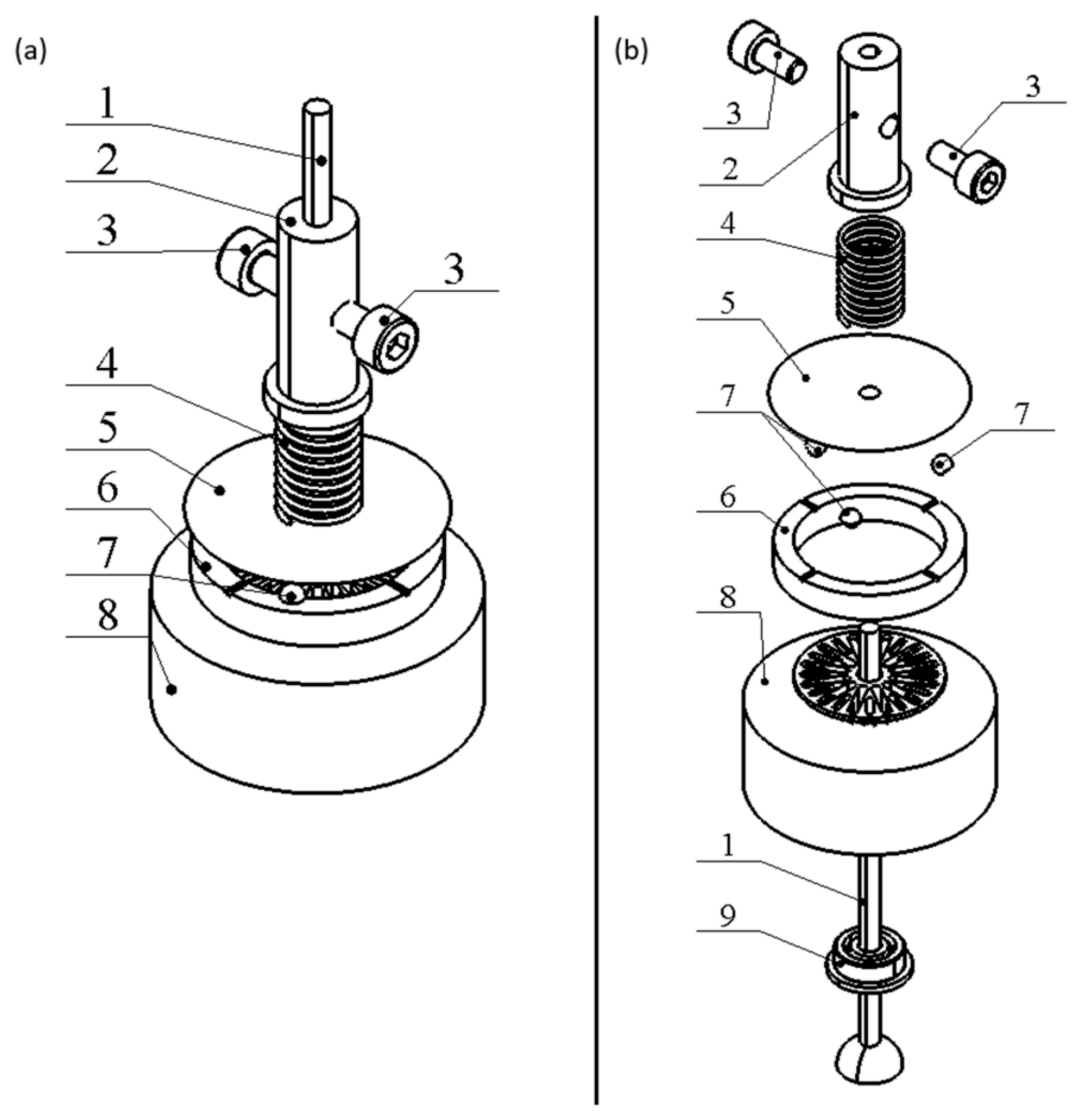
Structural design of the piezoelectric angular motion motor: (**a**)—assembled view; (**b**)—exploded view; 1—shaft; 2—preload spring clamping element; 3—bolts; 4—preload spring; 5—rotor; 6—piezoelectric ring; 7—spherical contact elements; 8—motor body; 9—ball bearing.

This direct-drive arrangement eliminates the need for gears or additional motion-conversion mechanisms and reduces the overall dimensions and structural complexity of the actuator. The assembled and exploded views of the proposed motor are presented in Figure 1.

As shown in Figure 1, the cylindrical motor body serves as the main supporting structure and provides a common reference axis for all mechanical components. The piezoelectric ring is mounted on the upper surface of the motor body and forms the active component of the stator. Three spherical contact elements are positioned between the piezoelectric ring and the cone-shaped rotor (5). These elements form discrete frictional interfaces through which the vibrational motion of the stator is transmitted to the rotor. The use of three contact elements provides a geometrically defined stator–rotor interface and ensures stable support of the rotor. In comparison with a continuous annular contact surface, discrete spherical contacts localize the interaction between the stator and rotor at predetermined points. This facilitates the generation and transmission of tangential motion while reducing the influence of small deviations in the flatness or parallelism of the contacting components. The spherical geometry also enables the contact elements to accommodate small relative inclinations between the stator and rotor surfaces. The rotor is rigidly connected to the output shaft, allowing the generated angular motion to be transmitted directly to an external mechanism. The lower part of the shaft is supported by a ball bearing integrated into the motor body. The bearing maintains the coaxial alignment of the rotor and shaft, limits radial displacement, and reduces mechanical resistance during angular motion.

Stable frictional contact between the rotor and spherical contact elements is maintained by a spring-based preload mechanism. The compression spring is positioned above the rotor and applies an axial force along the central axis of the motor. The spring is compressed using the clamping element, which is fixed to the shaft by the bolts. Variation in the spring compression changes the normal force acting at the three contact interfaces. Therefore, the proposed design allows the stator–rotor preload to be adjusted during assembly as well as operation. The preload force is an important parameter in the operation of a friction-driven piezoelectric motor.

The preload, in this study, was determined from the compression of the spring according to the following formula:

(1) $$F_{p}=k∆x$$

Here, *k* is the spring stiffness; ∆x is the spring compression.

The spring stiffness was *k* ≈ 0.65 N/mm (the spring stiffness was experimentally determined from the slope of the measured force–compression characteristic), while the experimentally selected compression of ∆x ≈ 3 mm corresponded to an axial preload of approximately 1.95 N. The preload setting was initially determined experimentally by gradually adjusting the spring compression until stable and continuous rotor operation without observable slipping was obtained. After this operating condition was identified, the spring compression was fixed, and the corresponding preload was maintained unchanged throughout all subsequent experiments.

If the applied preload is insufficient, intermittent contact or slipping may occur between the spherical contacts and the rotor, resulting in unstable rotational motion and reduced torque. In contrast, excessive preload increases mechanical damping and friction losses and may suppress the stator vibration amplitude. The spring-based mechanism was selected to provide a controllable and approximately constant axial force while preserving the compact coaxial arrangement of the motor.

**Figure 2:**
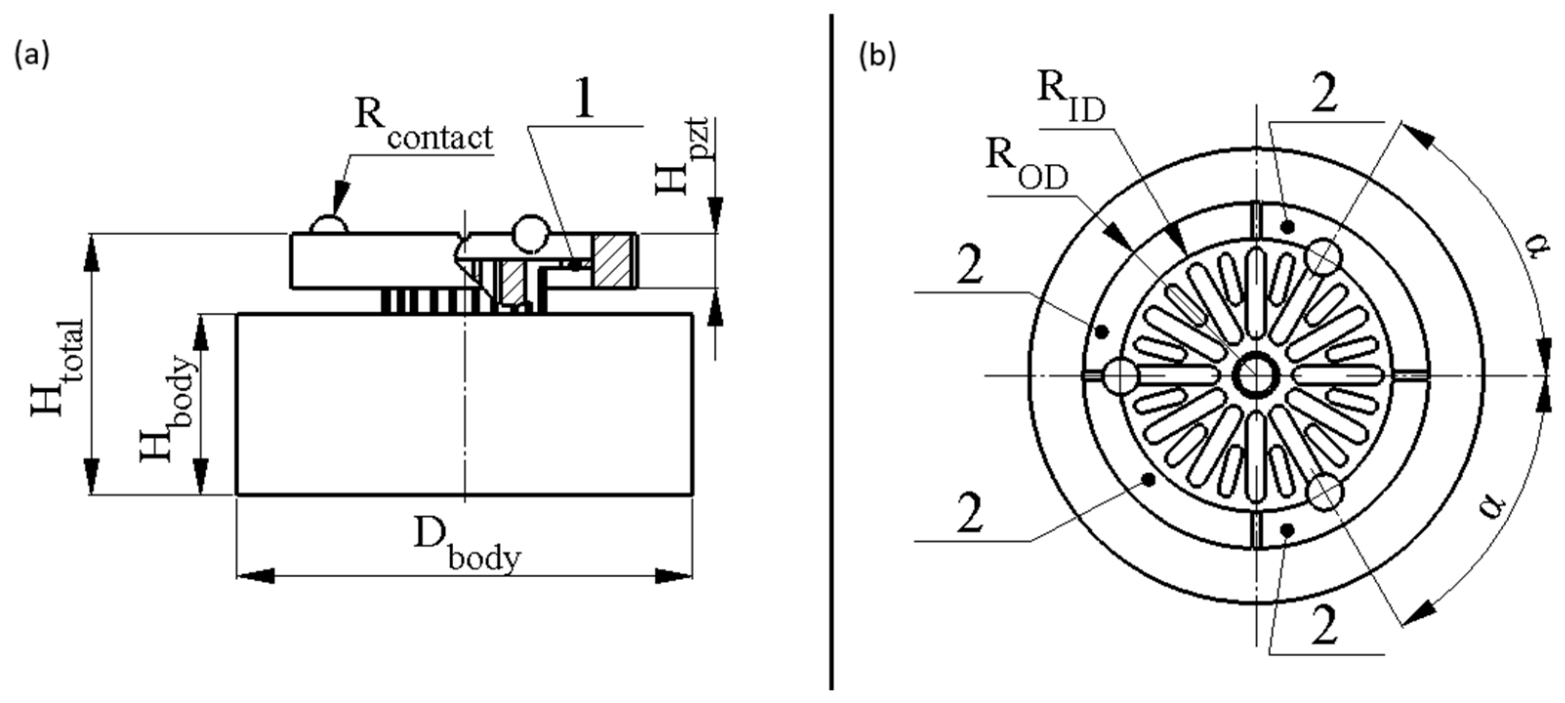
Geometrical characteristics of the stator: (**a**)—front view; (**b**)—top view; 1—clamping plate; 2—electrode.

The geometrical configuration of the stator, including the principal dimensions and the distribution of the contact elements, is shown in Figure 2.

The stator consists of a cylindrical supporting body, an annular piezoelectric ring, a clamping plate, and three spherical contact elements. The overall stator height, $H_{total}$ is 14.50 mm, while the height and diameter of the stator body, $H_{body}$ and $D_{body}$ are 10.00 mm and 25.00 mm, respectively. The piezoelectric ring has a height $H_{pzt}$ of 3.00 mm. The inner and outer dimensions of the ring, denoted by $R_{ID}$ and $R_{OD}$ are 7.50 mm and 9.50 mm, respectively. Each spherical contact has a radius $R_{contact}$ of 1.00 mm.

The three spherical contacts are symmetrically distributed around the central axis of the stator. Their positions are defined by the angular parameter α=60°, resulting in an angular separation of 120° between closest contact points. Such a distribution ensures that the rotor is supported at three circumferentially separated locations and that the tangential forces generated at the contact interfaces contribute to rotation about the central axis.

**Figure 3:**
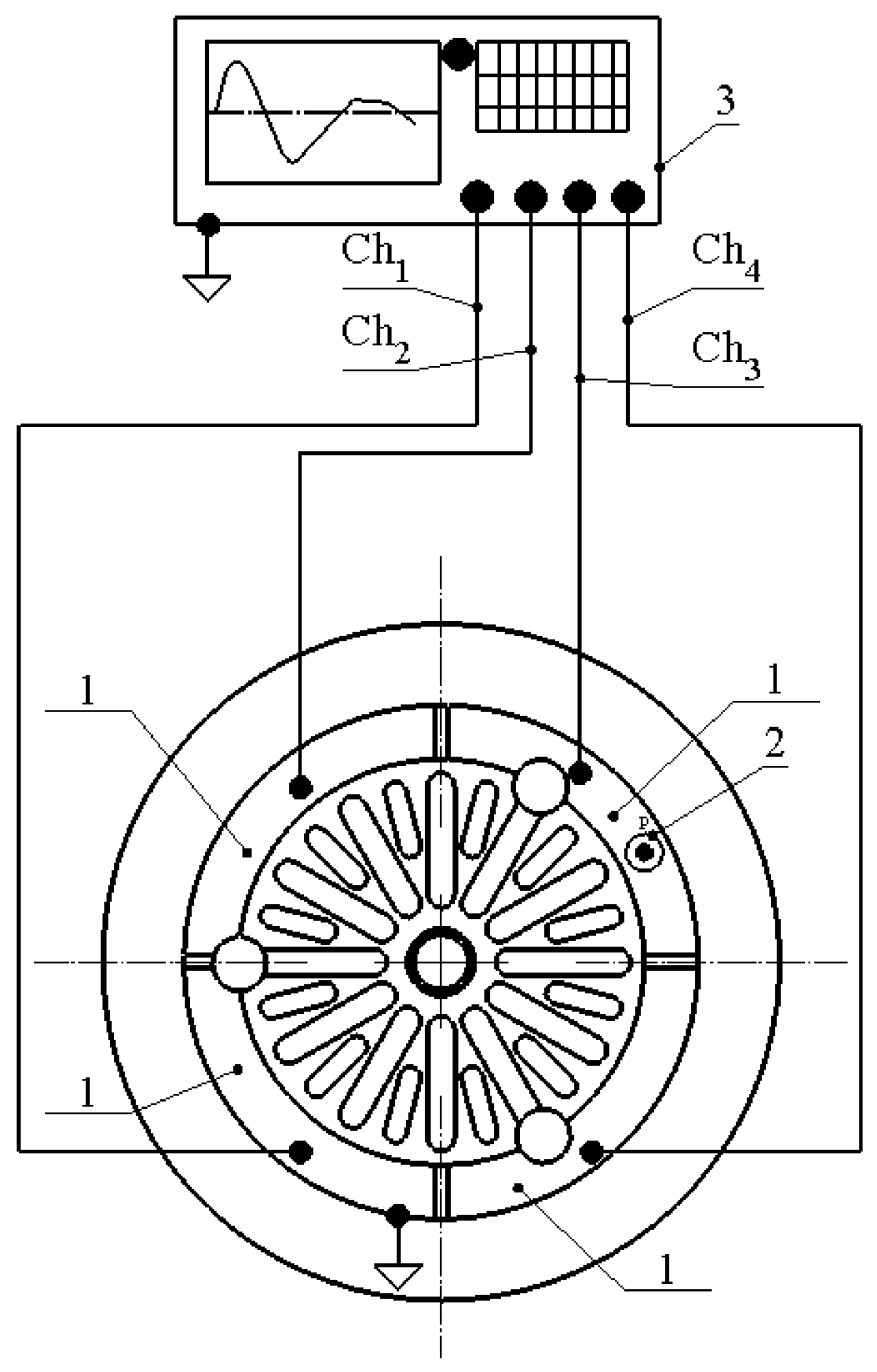
Excitation scheme of the motor: 1—electrodes on the upper surface of the piezoelectric ring; 2—polarization direction of the piezoelectric ring; 3—signal generator; ${Ch}_{1}{-Ch}_{4}$ —excitation-signal channels.

The piezoelectric ring is divided into four electrically isolated electrode regions located on its upper surface. The electrodes are circumferentially distributed around the ring. This configuration enables the individual electrode sections to be excited by independent harmonic excitation signals. The opposite surface of the piezoelectric ring is used as a common neutral electrode. When an excitation signal is applied to one of the upper electrodes, the corresponding region of the piezoelectric ring undergoes periodic deformation due to the inverse piezoelectric effect. Because the piezoelectric ring is mechanically coupled to the supporting body and clamping plate, its deformation excites a bending-type vibration of the complete stator. The spatial distribution of the electrodes is selected so that the individual electrode regions can excite different spatial components of the same circumferential vibration mode. The electrical excitation scheme used to generate the traveling wave in the stator is presented in Figure 3.

As shown in Figure 3, the four upper electrodes are connected to four independent channels of the signal generator. The lower common electrode is set as neutral. All channels operate at the same excitation frequency and nominal voltage amplitude, while the phases of the applied signals are shifted relative to one another. For an ideal four-phase excitation scheme, the voltage signals can be expressed as:

(2) $${Ch}_{1}\left(t\right)=U_{0}\sin\left(\omega t\right);$$

(3) $${Ch}_{2}\left(t\right)=U_{0}\sin\left(\omega t+\frac{\pi }{2}\right);$$

(4) $${Ch}_{3}\left(t\right)=U_{0}\sin\left(\omega t+\pi \right);$$

(5) $${Ch}_{4}\left(t\right)=U_{0}\sin\left(\omega t+\frac{3\pi }{2}\right).$$

where $U_{0}$ is the excitation voltage amplitude, ω is the angular excitation frequency, and t is time; *Ch*_1_…*Ch*_4_ are excitation channels.

The phase shift between consecutive channels produces a progressive deformation of the piezoelectric ring around its circumference. Instead of all stator regions reaching their maximum displacement simultaneously, the position of the maximum deformation continuously moves from one electrode region to the next. When the excitation frequency is close to the resonant frequency of the selected stator mode, the superposition of these spatially and temporally shifted deformations generates a circumferential traveling wave. The traveling wave causes points on the stator surface to follow elliptical trajectories. At each spherical contact location, the local displacement can be resolved into normal and tangential components relative to the rotor surface. The normal displacement component periodically changes the contact force between the stator and rotor, while the tangential component produces a friction force acting along the circumference of the rotor. As the traveling wave propagates around the stator, the tangential motion of the three spherical contacts transfers stator vibrations to the rotor and generates continuous angular motion.

The rotor rotation direction is opposite to the tangential motion of the stator surface at the active contact interface and depends on the propagation direction of the traveling wave. Reversing the sequence of the phase-shifted excitation signals reverses the wave propagation direction and consequently changes the direction of rotor rotation. Therefore, bidirectional angular motion can be achieved without changing the mechanical configuration of the motor. The rotational performance of the motor is governed by the interaction between the stator vibration and the frictional contact conditions. The excitation frequency determines the proximity to the resonant state, while the excitation signal amplitude influences the stator vibration amplitude and the tangential displacement of the spherical contacts. The preload force affects the normal contact force and therefore the amount of tangential force that can be transferred to the rotor. Consequently, the output speed and torque depend on the excitation frequency, voltage amplitude, phase relationship between the signals, friction coefficient, and applied preload.

The proposed construction of the motor combines a single ring-shaped piezoelectric element, four-channel traveling-wave excitation, and three discrete spherical contacts in a compact coaxial arrangement. The operating principle relies on transforming the electrically induced vibration of the piezoelectric ring into elliptical motion at the contact points and subsequently into continuous rotation of the rotor and output shaft. To determine the vibration mode suitable for traveling-wave generation and evaluate the displacement characteristics of the contact points, a numerical investigation of the stator was performed, as presented in the following section.

## 3. Numerical Investigation of the Angular Motion Motor

Numerical investigations were performed to verify the operating principle of the proposed motor and to determine its electromechanical characteristics. Particular attention was given to the identification of the operating vibration mode, analysis of the electrical impedance and phase characteristics, evaluation of the displacement components at the spherical contact points, and determination of their motion trajectories. The finite element model was built by COMSOL Multiphysics 6.1 software according to the geometrical characteristics of the motor presented in Figure 1 and Figure 2. The numerical model was limited to characterization of the electromechanical response of the stator assembly. The rotor, preload spring, and stator–rotor frictional contact interaction were not included in the model. Therefore, no preload force, friction, contact separation, slip, or contact stress was considered in the numerical analysis. A representative dynamic friction coefficient for the alumina-sphere/rotor interface was not identified experimentally and was therefore not prescribed in the numerical model. The spherical contact elements were included as structural components of the stator, and their displacement characteristics were evaluated under unloaded conditions.

The electrical boundary conditions and polarization direction of the piezoelectric ring were defined according to the excitation scheme shown in Figure 3. The lower mounting surfaces of the motor body were rigidly fixed by restricting displacement in all coordinate directions. The piezoelectric ring was defined as hard PZT-8 piezoceramic, while alumina oxide characteristics were assigned to spherical contact elements. The motor body was defined as made of acrylic plastic. The material characteristics used in the numerical model are summarized in Table 1.

**Figure 4:**
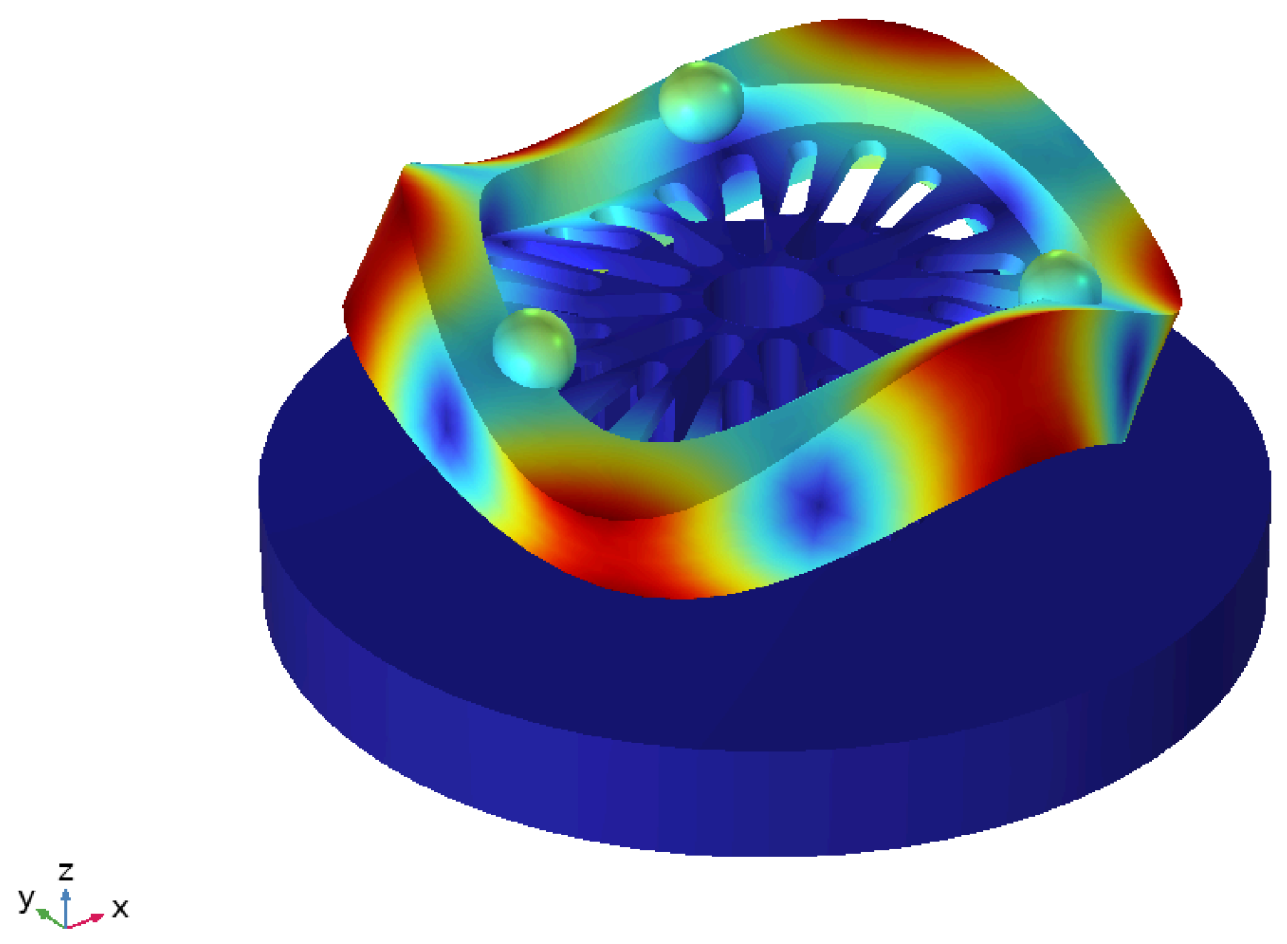
Modal shape of the motor stator at an eigenfrequency of 39.95 kHz.

After defining the material properties and boundary conditions, a modal analysis was performed to identify a vibration mode suitable for generating the traveling wave and transferring motion to the rotor through the spherical contacts. The identified stator modal shape and its eigenfrequency are presented in Figure 4.

The modal shape was obtained at an eigenfrequency of 39,952 Hz. The mode is characterized by a periodic circumferential bending deformation of the ring-shaped stator. Alternating regions of positive and negative out-of-plane displacement are distributed along the circumference, while the supporting motor body remains nearly stationary due to its considerably higher structural rigidity and the applied fixed constraint. Appreciable displacement is also generated at the locations of the three spherical contacts, confirming that the modal shape can be used to transfer vibrational motion to the rotor. The modal analysis represents a standing-wave solution. However, the circumferentially shifted electrode groups allow two spatial components of the selected mode to be excited with an appropriate temporal phase difference. Their superposition produces a traveling wave vibration along the circumference of the stator. Consequently, the spherical contacts experience both tangential and normal displacement components, which are required for friction-based angular motion generation.

**Figure 5:**
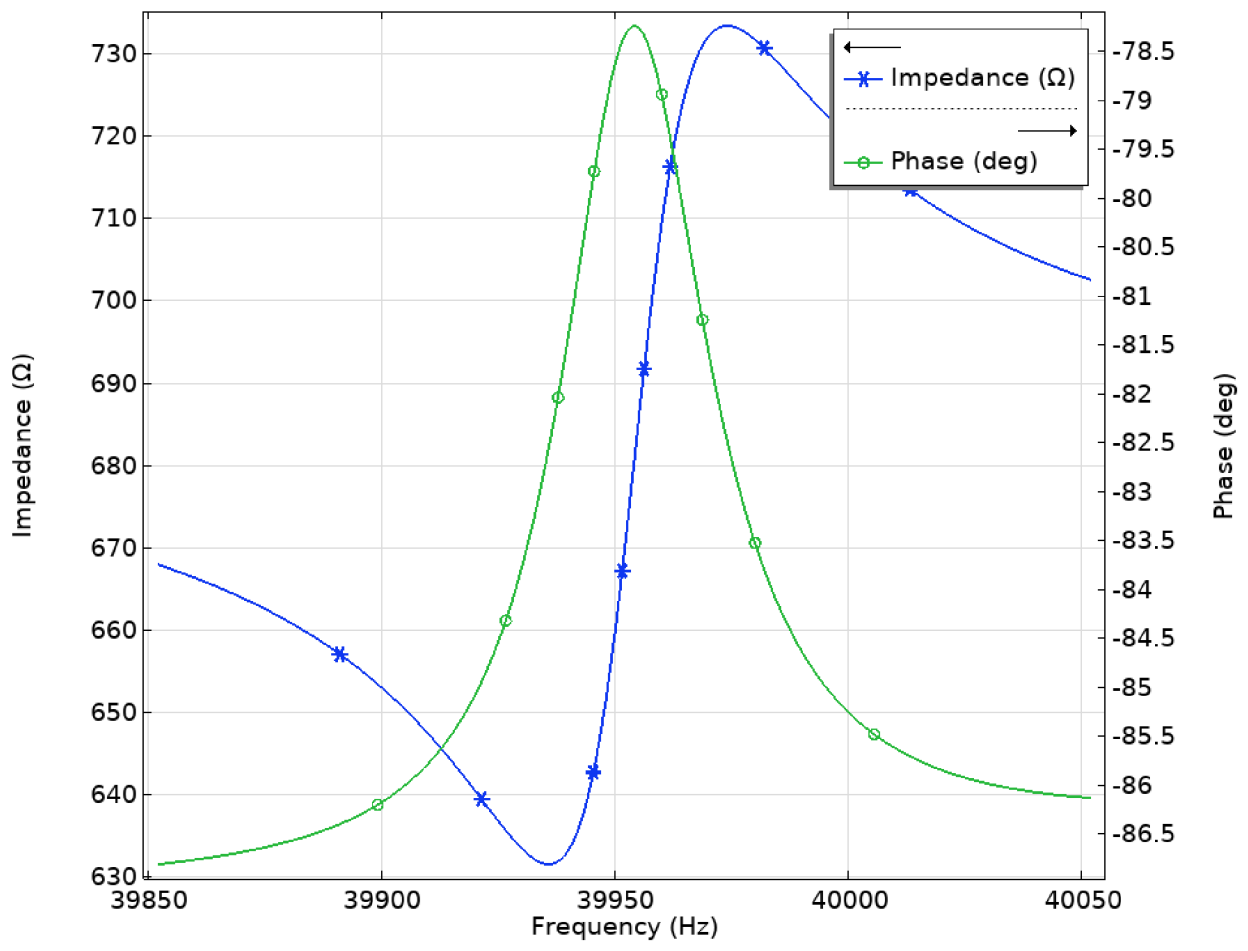
Numerically calculated electrical impedance and phase frequency characteristics of the motor stator.

To determine the electrical resonance region associated with the selected vibration mode, a frequency-domain analysis was subsequently performed. The calculated electrical impedance and phase characteristics are shown in Figure 5.

As can be observed in Figure 5, the resonance frequency of the motor was identified at approximately 39,936 Hz, while the antiresonance frequency was obtained at approximately 39,974 Hz. A slight mismatch of 16 Hz, or approximately 0.04%, was found between the resonance frequency and the eigenfrequency of 39,952 Hz obtained from the modal analysis. This small difference appears due to discrete steps used in the frequency domain study. However, the agreement between modal and frequency domain studies can be assessed as good. Furthermore, the effective electromechanical coupling coefficient *k_eff_* of the motor was calculated using Equation (6) and was found to be 4.36%.

(6) $$\frac{k_{eff}^{2}}{1-k_{eff}^{2}}=\frac{f_{a}^{2}-f_{r}^{2}}{f_{r}^{2}}.$$

Here, *k_eff_* is the electromechanical effective coupling; $f_{a}$ is the antiresonance frequency; and $f_{r}$ is the resonance frequency.

The obtained impedance and phase characteristics confirmed that the modal shape can be excited within a narrow frequency range around 39.95 kHz. However, electrical resonance alone does not fully describe the suitability of the stator for generating angular motion. The mechanical response at the spherical contact points must also provide sufficiently large tangential and normal displacement components, as their combined action determines the motion transmitted to the rotor.

**Figure 6:**
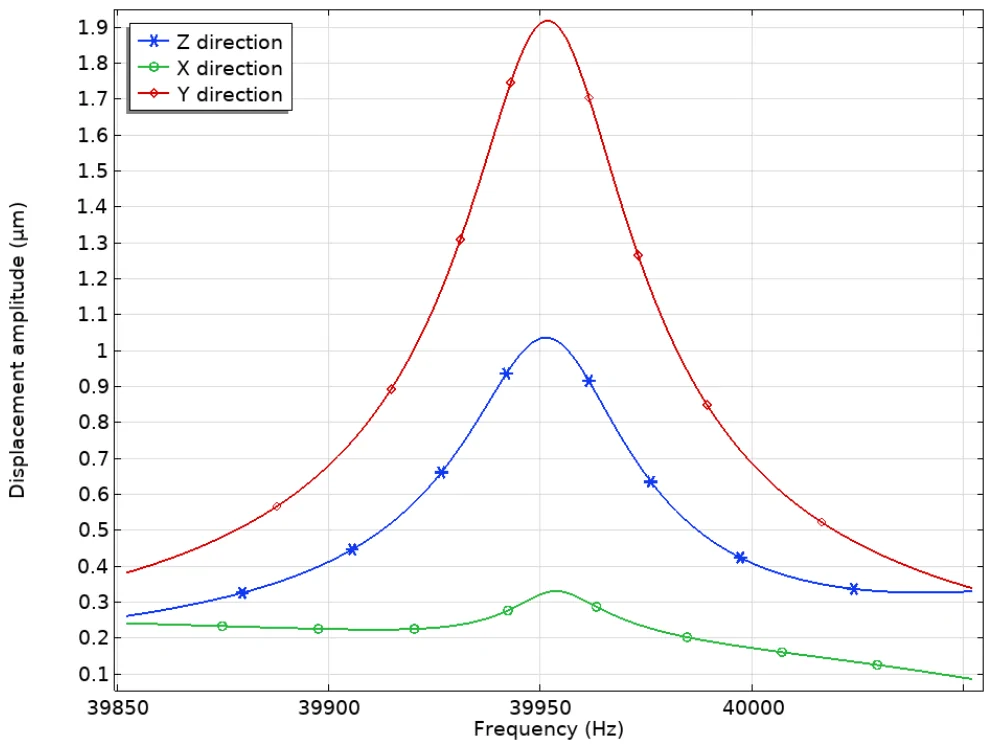
Displacement amplitudes of spherical contacts in the X, Y, and Z directions.

Therefore, the next stage of the numerical investigation was focused on the displacement–frequency characteristics of a spherical contact point. The displacement amplitudes in the X, Y, and Z directions were calculated over the frequency range covering the resonance and antiresonance region. This analysis enabled the dominant displacement direction to be identified and the frequencies corresponding to the maximum mechanical response to be compared with the electrical and modal analysis results. The calculated displacement-frequency characteristics are presented in Figure 6.

As can be observed in Figure 6, all three displacement components reached their maximum values within a very narrow frequency interval from 39,951 to 39,954 Hz. This interval corresponds closely to the natural frequency obtained from the modal analysis and lies between the resonance and antiresonance frequencies identified from the electrical characteristics. Such agreement confirms that the maximum mechanical response is associated with the selected operating vibration mode. The highest displacement amplitude was obtained in the Y direction and reached 1.91 μm at 39,952 Hz. The maximum Z-component was 1.03 μm at 39,951 Hz, while the X-component reached 0.32 μm at 39,954 Hz. Therefore, the Y-direction displacement was approximately 1.85 times higher than the Z-direction displacement and almost six times higher than the X-direction displacement. For the investigated contact element, the Y-component represents displacement tangential to the nominal stator–rotor interface, while the Z-component represents displacement normal to this interface. Under assembled operating conditions, these displacement components provide the kinematic conditions required for friction-based motion transmission. However, the corresponding frictional and normal contact forces are not calculated in the present model. Although the X-direction displacement was considerably smaller, it indicates that the deformation of the stator is spatial and not restricted to a single plane. Nevertheless, the Y- and Z-components have the primary influence on the generation of angular motion. The similar shapes of the displacement–frequency curves and the close locations of their maxima indicate that all three displacement components originate from the same coupled resonant mode. A rapid reduction in displacement was observed when the excitation frequency moved away from approximately 39.95 kHz, demonstrating that the mechanical response of the stator is strongly frequency dependent. Therefore, precise selection of the excitation frequency is essential for maximizing the motion transferred through the spherical contacts.

**Figure 7:**
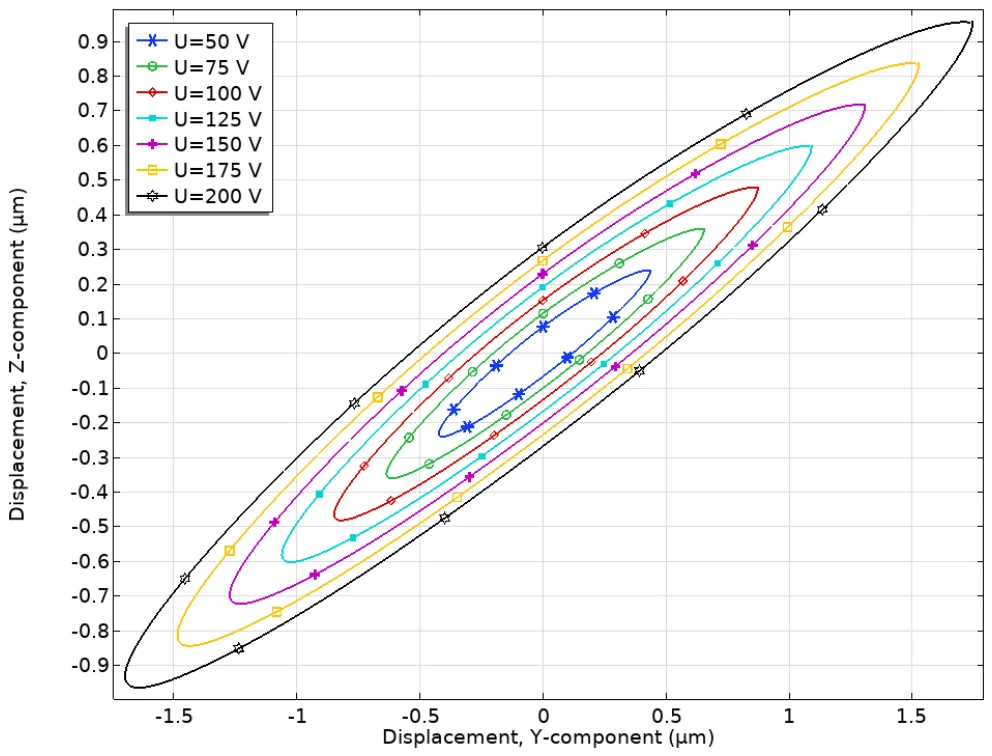
Numerically calculated trajectories of the unloaded spherical contact in the Y-Z plane at different excitation voltages.

However, the displacement amplitudes alone do not indicate whether the tangential and normal components occur simultaneously or with a phase difference. To evaluate the actual motion of the contact point under unloaded conditions during one excitation period, its trajectory was calculated in the Y-Z plane at excitation voltages ranging from 50 to 200 V_p-p_. The obtained trajectories are presented in Figure 7.

As shown in Figure 7, closed elliptical displacement trajectories were obtained for all investigated excitation voltages. The elliptical shape confirms that the tangential Y-component and normal Z-component of the stator displacement are phase shifted, which is a necessary kinematic condition for traveling-wave friction-driven operation. However, because the rotor, preload, and frictional contact interaction were not included in the numerical model, these trajectories represent the unloaded motion of the spherical contact elements rather than their actual trajectories under operating preload. The inclination of the trajectories indicates that the tangential and normal displacement components are strongly coupled. During one part of the excitation cycle, the spherical contact element moves tangentially while its normal displacement is directed toward the nominal rotor interface, whereas during the opposite part of the cycle the tangential motion reverses and the normal displacement changes accordingly. An increase in excitation voltage from 50 to 200 V_p-p_ resulted in a continuous enlargement of the elliptical trajectories. At 200 V_p-p_, the displacement range reached approximately 3.5 μm in the Y direction and 1.9 μm in the Z direction. The orientation and general shape of the trajectories remained nearly unchanged with increasing voltage, indicating preservation of the spatial character and phase relationship of the selected vibration mode. The approximately proportional increase in trajectory dimensions indicates an essentially linear vibration response within the investigated excitation range. Therefore, the excitation voltage can be used to control the magnitude of the contact-point motion. The obtained elliptical trajectories confirm the suitability of the vibration mode and excitation scheme for generating continuous angular motion.

The numerical investigation confirmed that the proposed stator configuration is suitable for generating traveling-wave angular motion. The selected operating mode was obtained at approximately 39.95 kHz and showed good agreement with the resonance region identified from the electrical impedance and phase characteristics. The calculated mismatch between the natural and resonance frequencies was only 16 Hz, corresponding to approximately 0.04%, while the effective electromechanical coupling coefficient was *k_eff_* = 4.36%.

The displacement analysis showed that the motion of the spherical contact elements contains dominant tangential and normal components with the phase relationship required for elliptical trajectories. These unloaded trajectories were maintained over the investigated excitation range from 50 to 200 V_p-p_, while their dimensions increased with excitation voltage. The numerical analysis therefore characterizes the kinematic and electromechanical conditions required for traveling-wave operation but does not represent the nonlinear stator–rotor interaction under preload.

Overall, the numerical investigation identified the appropriate operating vibration mode and resonance and demonstrated the formation of the required phase-shifted tangential and normal displacement components. The actual frictional transmission of these vibrations to the rotor was subsequently evaluated experimentally. Detailed modeling of the preloaded stator–rotor interface, including friction, possible separation, slip, and local contact stress, is beyond the scope of the present study and will be addressed in future work.

## 4. Experimental Investigation of the Angular Motion Motor

**Figure 8:**
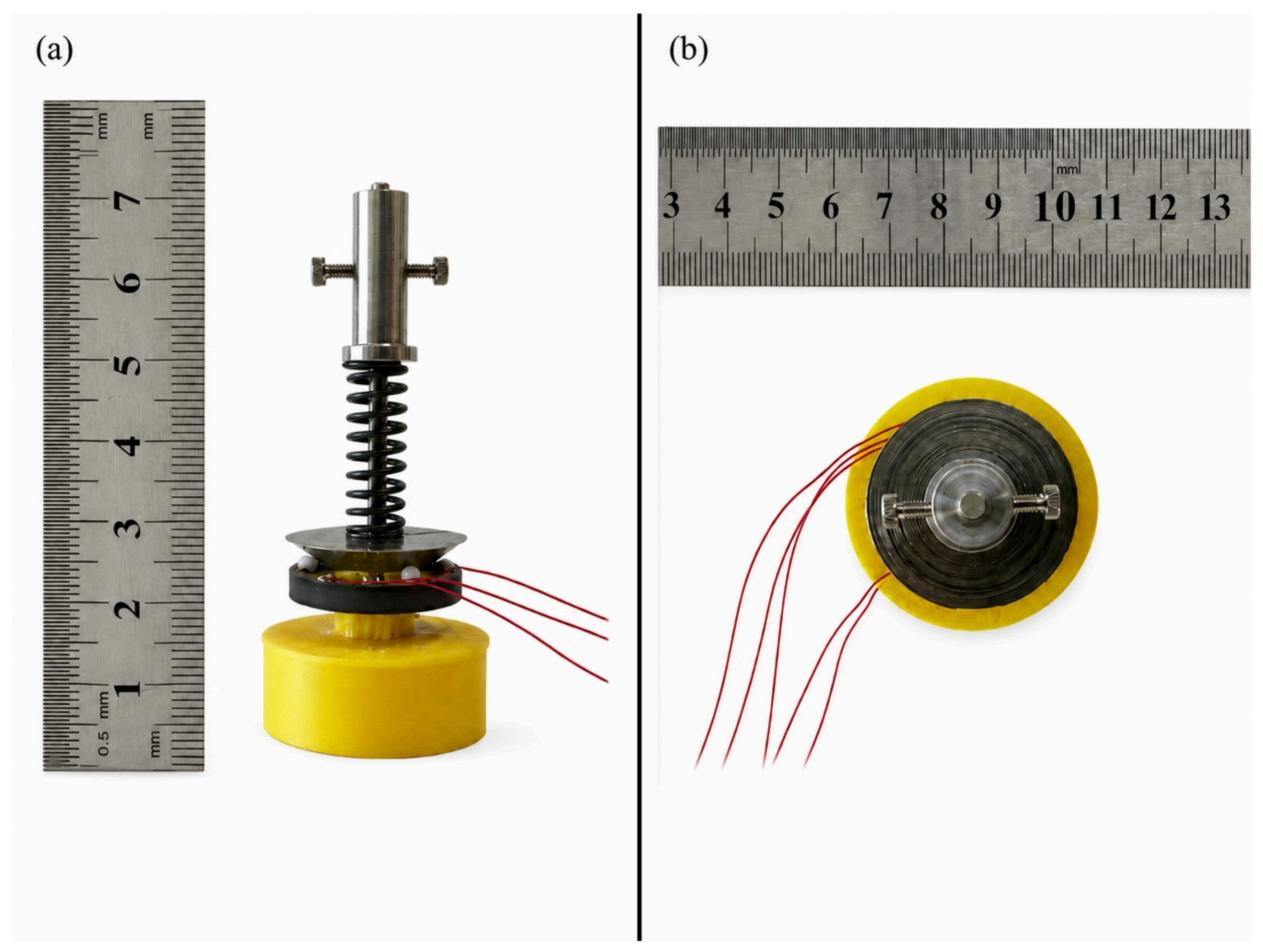
Prototype of the proposed piezoelectric angular motion motor: (**a**)—side view; (**b**)—top view.

Experimental investigations were conducted to verify the operating principle of the proposed motor and to evaluate its electromechanical and mechanical characteristics. A prototype was manufactured according to the geometrical parameters presented in Figure 1 and Figure 2, the excitation configuration shown in Figure 3, and the material properties summarized in Table 1. Side and top views of the fabricated motor prototype are presented in Figure 8.

**Figure 9:**
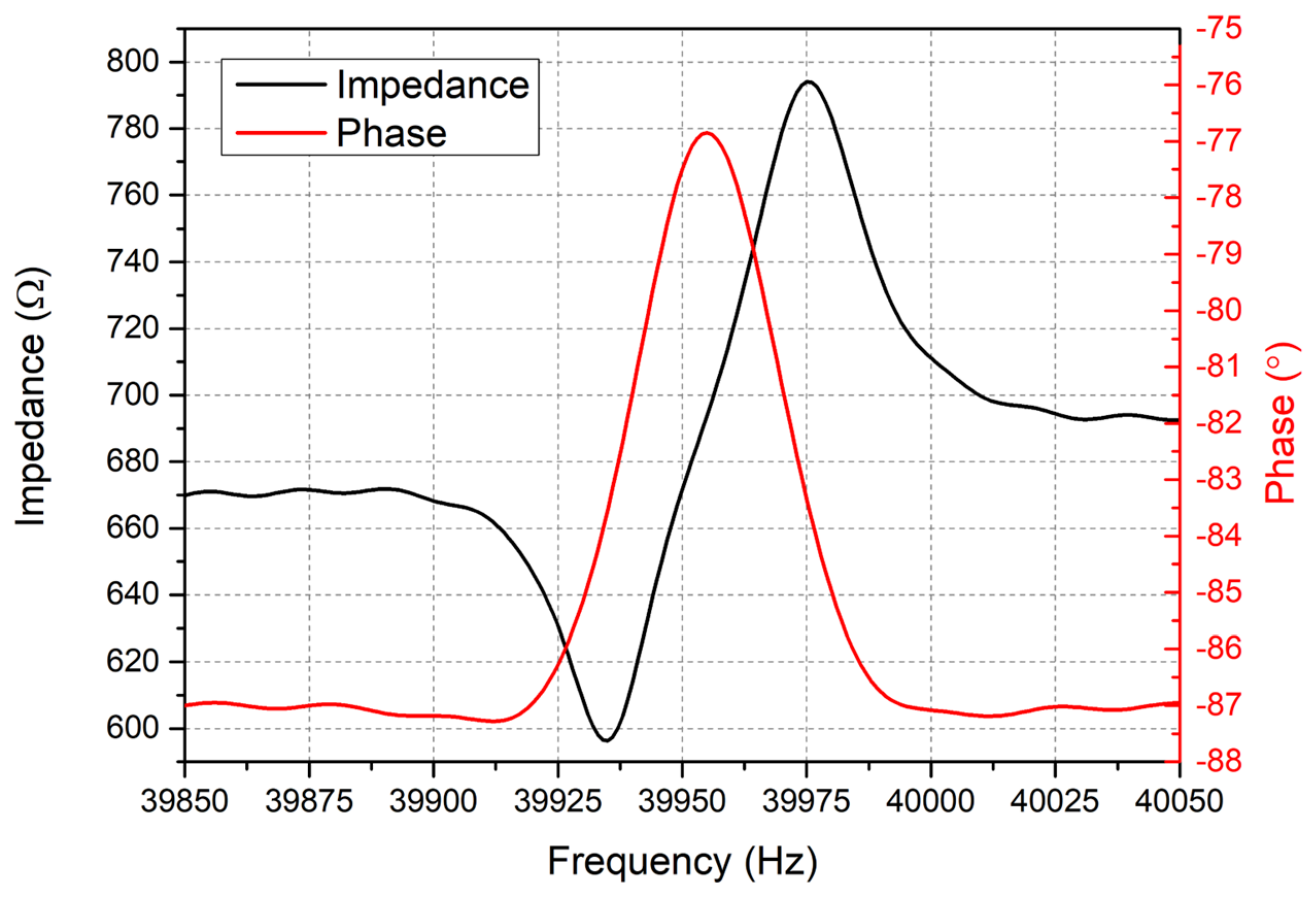
Impedance– and phase–frequency characteristics of the angular motion motor.

The experimental investigation of the prototype (Figure 8) was performed in several stages. First, the impedance- and phase-frequency characteristics of the fabricated piezoelectric motor were measured to identify the resonance and antiresonance frequencies and to compare them with the numerical results. The measurements were carried out using a SinePhase 16777k impedance analyzer (SinePhase, Mödling, Austria). The experimentally obtained impedance and phase characteristics are presented in Figure 9.

As can be observed in Figure 9, the resonance frequency of the prototype was identified at 39,935 Hz, while the antiresonance frequency was obtained at 39,975 Hz. The experimentally measured minimum impedance at resonance was 596.28 Ω, whereas the maximum impedance at antiresonance reached 793.98 Ω. In comparison, the corresponding numerically calculated impedance values were approximately 631.6 Ω and 733.2 Ω, respectively. Therefore, the measured resonance impedance was approximately 35.3 Ω, or 5.6%, lower than the numerical value, while the measured antiresonance impedance was approximately 60.8 Ω, or 8.3%, higher.

The experimentally determined resonance frequency differed from the numerically calculated value of 39,936 Hz by only 1 Hz, corresponding to approximately 0.003%. A similarly small difference was obtained for the antiresonance frequency, as the numerical and experimental values were 39,974 and 39,975 Hz, respectively. Furthermore, the effective electromechanical coupling coefficient calculated from the experimental resonance and antiresonance frequencies was *k_eff_* = 4.47%, while the numerical value was 4.36%. The difference between the calculated coupling coefficients is approximately 2.5%.

Although larger differences were observed between the measured and calculated impedance magnitudes than between the corresponding characteristic frequencies, the shapes of the impedance and phase curves and the locations of the resonance and antiresonance regions were in good agreement. The differences in impedance magnitude can be associated with manufacturing tolerances, variations in the material properties of the fabricated components, mechanical losses, electrical connection resistance, and damping effects that were only approximately represented in the numerical model. Overall, the experimental results confirmed that the operating vibration mode of the prototype was located within the frequency range predicted by the numerical investigation.

**Figure 10:**
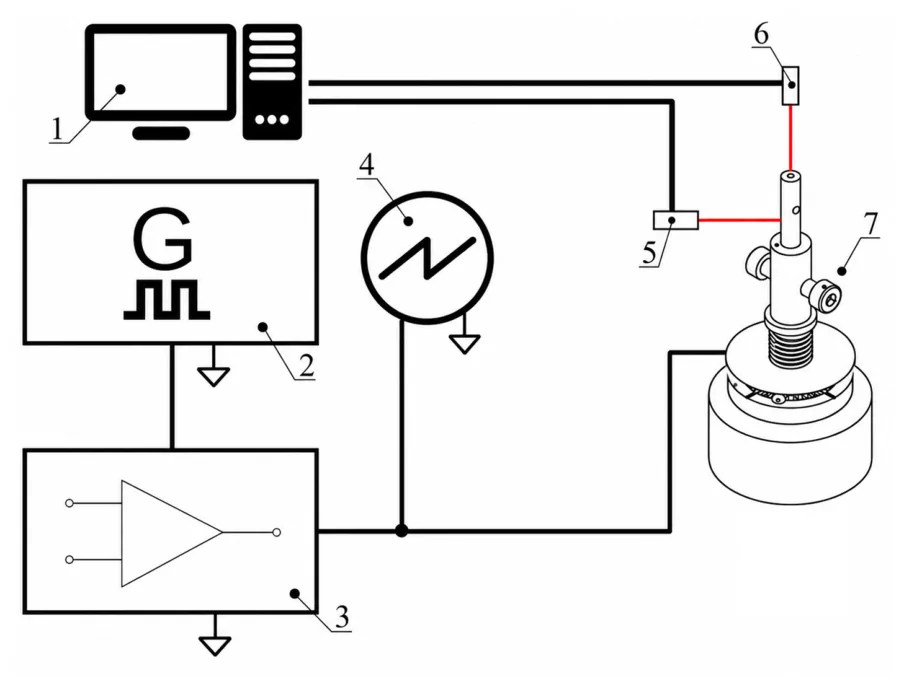
Schematic of experimental setup; 1—a computer with data acquisition software; 2—signal generator; 3—power amplifier; 4—oscilloscope; 5—non-contact displacement sensor; 6—non-contact tachometer; 7—a prototype of the motor.

In order to perform experimental investigations of the motor, an experimental setup was assembled. A schematic of the experimental setup is shown in Figure 10.

Therefore, the experimental setup was based on a computer with data acquisition software, a function generator WW5064 (Tabor Electronics, Nesher, Israel), a power amplifier PX-200 (Piezo Drive, Shortland, Australia), an oscilloscope DL2000 (Yokogawa, Tokyo, Japan), a non-contact tachometer CA 1727 (Chauvin Arnoux, Paris, France), and a non-contact displacement sensor optoNCDT 1420 (Micro-Epsilon, Ortenburg, Germany).

For each excitation amplitude, four independent measurements were performed, and the reported values represent the mean values. Error bars represent the standard deviation. The excitation frequency was fixed at 39.935 kHz, corresponding to the experimentally determined resonance frequency. Rotational-speed and incremental-motion measurements were performed under no external mechanical load.

During all experimental investigations, including rotational-speed, stall-torque, and incremental-motion measurements, the axial preload was fixed at approximately 1.95 N and was not changed between individual tests.

**Figure 11:**
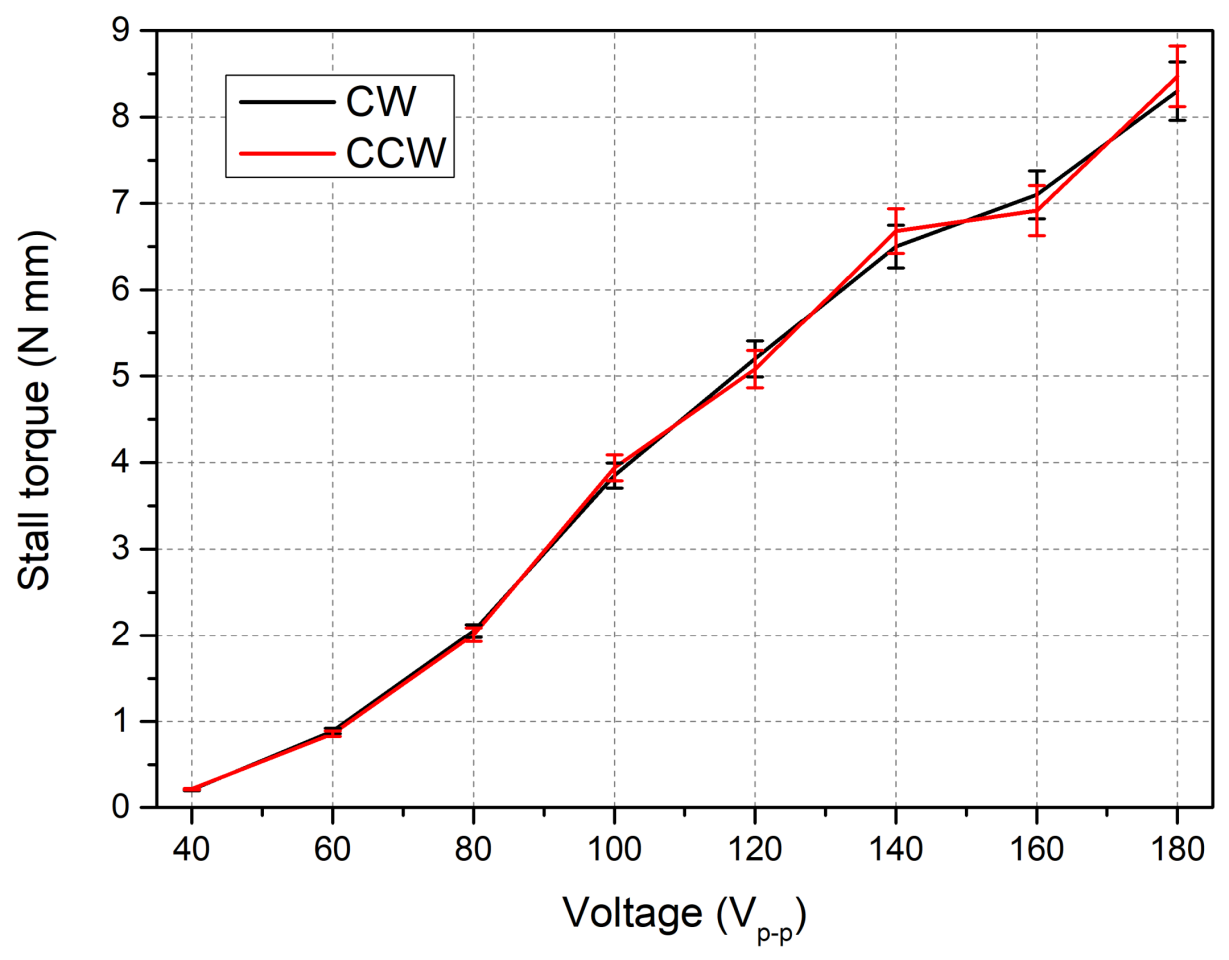
Stall torque characteristics of motor under different excitation signal amplitudes.

The stall torque of the motor was measured using a PCE-DFG N 10 digital force gauge (PCE Deutschland GmbH, Meschede, Germany) and a short lever arm rigidly attached to the motor shaft. The force sensor was positioned to measure a tangential force at a known distance from the rotation axis. Measurements were performed for both clockwise (CW) and counterclockwise (CCW) rotation with excitation signal amplitudes from 40 V_p-p_ to 180 V_p-p_ with an increment step of 20 V_p-p_. Results of measurements are given in Figure 11.

The stall-torque characteristics obtained for CW and CCW rotation are shown in Figure 11. The stall torque increased monotonically with excitation-signal amplitude, from approximately 0.2 N·mm at 40 V_p-p_ to approximately 8.3–8.4 N·mm at 180 V_p-p_. The CW and CCW characteristics followed nearly identical trends over the entire investigated voltage range, and the differences between the two directions remained within the experimental variation. This indicates that reversal of the traveling-wave propagation direction does not substantially affect the torque-transmission capability of the stator–rotor interface. The increasing experimental variation at higher excitation amplitudes may be associated with the greater influence of nonlinear friction and contact conditions. Overall, the results confirm comparable stall-torque performance in both rotational directions and provide additional experimental validation of the bidirectional operation of the proposed motor.

**Figure 12:**
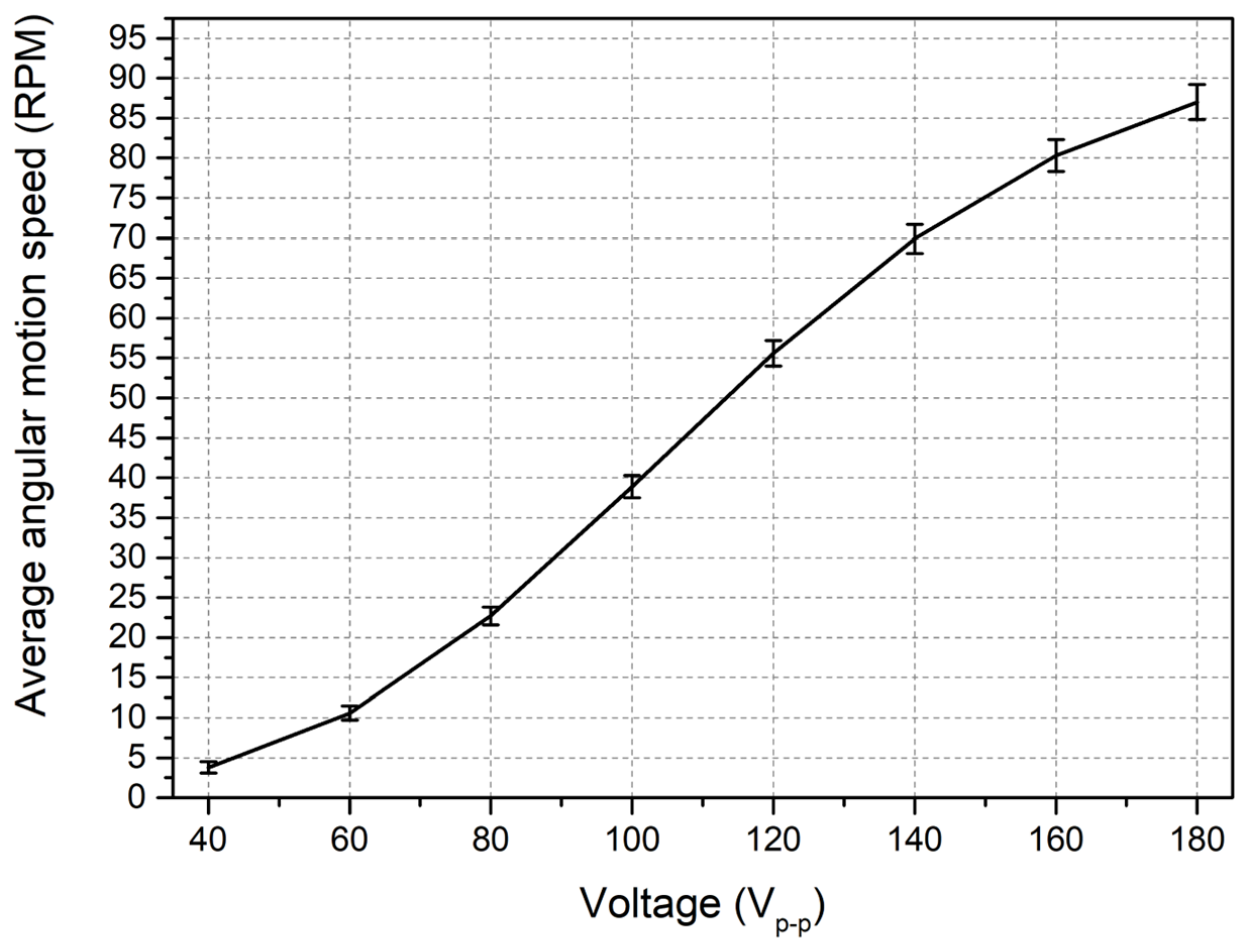
Angular motion characteristics of the motor under different excitation signal amplitudes.

The next stage of the experimental investigation was dedicated to the indication of the angular motion characteristics of the motor at different excitation-signal amplitudes. The excitation signal amplitude was varied from 40 to 180 V_p-p_ in increments of 20 V_p-p_, while the excitation frequency and the remaining experimental conditions were kept unchanged. For each excitation signal amplitude, repeated rotational-speed measurements were performed, and the mean values and corresponding standard deviations were calculated. The obtained results are presented in Figure 12.

As can be observed in Figure 12, the rotational speed increased continuously with increasing excitation-signal amplitude. At the lowest investigated voltage of 40 V_p-p_, the motor reached a mean rotational speed of 3.8 RPM, with a standard deviation of 0.7 RPM. Increasing the excitation voltage to 60 and 80 V_p-p_ increased the rotational speed to 10.6 ± 0.9 RPM and 22.7 ± 1.1 RPM, respectively. The most pronounced increase in rotational speed was observed within the intermediate excitation range. At 100 and 120 V_p-p_, the measured speeds were 38.9 ± 1.4 RPM and 55.6 ± 1.6 RPM, respectively. A further increase in the excitation voltage to 140 and 160 V_p-p_ resulted in rotational speeds of 69.9 ± 1.8 RPM and 80.3 ± 2.0 RPM. The maximum rotational speed of 87.0 ± 2.2 RPM was obtained at 180 V_p-p_.

The obtained relationship between excitation voltage and rotational speed was nonlinear. At low excitation amplitudes, the displacement of the spherical contacts was relatively small, and a considerable part of the generated tangential force was required to overcome static friction and mechanical resistance within the rotor system. Therefore, the increase in rotational speed was comparatively limited between 40 and 60 V_p-p_. Within the intermediate voltage range of approximately 80–140 V_p-p_, an increase in the vibration amplitude of the stator produced a substantial enlargement of the elliptical contact-point trajectories. Consequently, the tangential displacement and the mechanical energy transferred to the rotor increased, resulting in a more rapid rise in rotational speed. This trend is consistent with the numerical results, which showed that the dimensions of the spherical-contact trajectories increased with excitation voltage. At excitation voltages above approximately 140 V_p-p_, the rate of increase in rotational speed gradually decreased. The speed increased by 10.4 RPM between 140 and 160 V_p-p_, but only by 6.7 RPM between 160 and 180 V_p-p_. This tendency indicates the onset of a saturation region, which can be associated with increased slipping at the stator–rotor interface, mechanical losses, nonlinear contact behavior, and a reduction in the effective transfer of additional vibration energy to the rotor.

The absolute standard deviation increased slightly from 0.7 RPM at 40 V_p-p_ to 2.2 RPM at 180 V_p-p_. However, the relative variation decreased from approximately 18.4% to 2.5%, indicating that the motor operation became more stable as the excitation voltage increased. Overall, the obtained results confirmed that the rotational speed of the motor can be controlled by varying the excitation-signal amplitude, while the maximum measured speed under the investigated conditions was 87 RPM.

The subsequent investigation focused on evaluating the resolution of the angular displacement of the motor under different excitation voltages. Experimental measurements of the angular resolution of the motor were performed using a non-contact displacement sensor optoNCDT 1420 (Micro-Epsilon, Ortenburg, Germany). The non-contact displacement sensor was directed toward a short lever arm rigidly connected to the motor shaft. The linear displacement ∆x measured at a known radial distance *L* of 5 mm from the shaft axis was converted into angular displacement according to

(7) $$\theta ={tan}^{-1}(\frac{∆x}{L})$$

**Figure 13:**
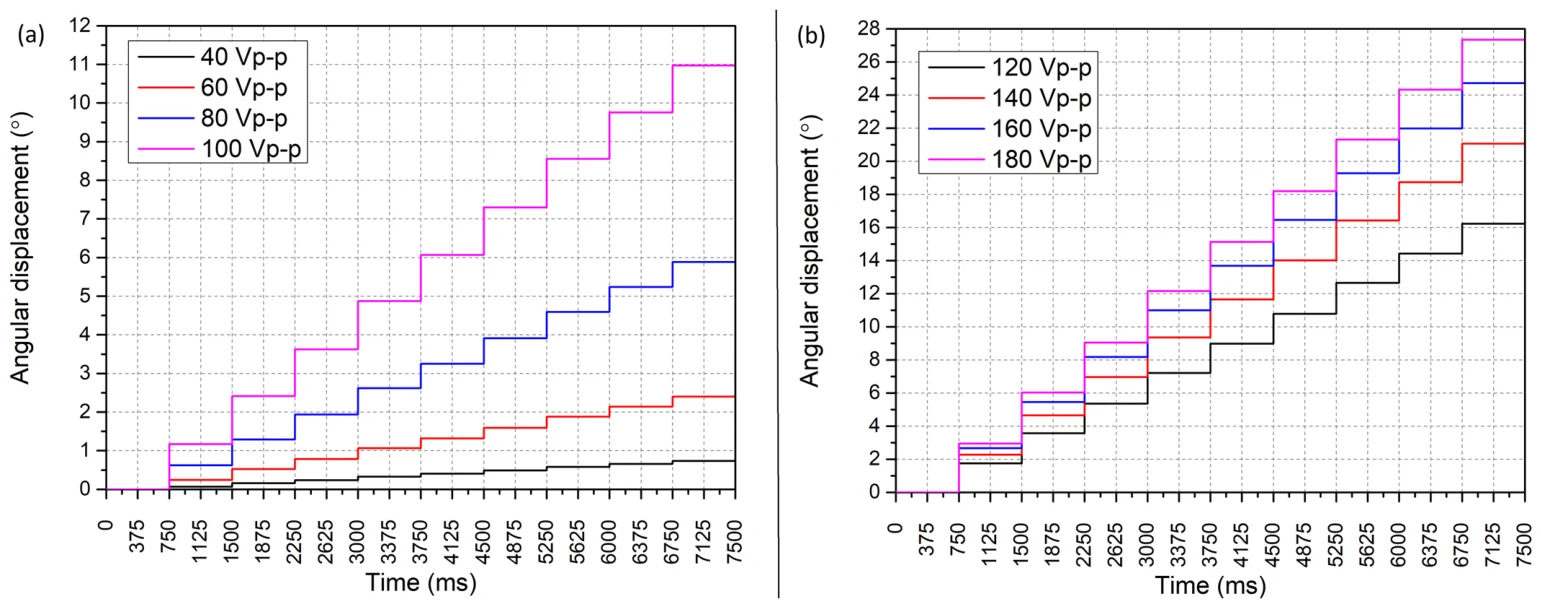
Angular motion resolution under different excitation signal amplitudes: (**a**) range from 40 V_p-p_ to 100 V_p-p_; (**b**) range from 120 V_p-p_ to 180 V_p-p_.

The burst-type sine excitation signal consisting of 15 cycles at the resonance frequency of the motor during a 750 ms one-step time period was used. These excitation parameters were indicated experimentally, i.e., it was indicated that 15 cycles are the minimal number of cycles necessary to obtain angular motion at a 40 V_p-p_ excitation signal amplitude, while 750 ms is the shortest time range that is necessary for proper displacement capture by the optoNCDT 1420 (Micro-Epsilon, Ortenburg, Germany) sensor. Therefore, these parameters were used to drive the motor, while this arrangement of the excitation signal ensures a stable stepping-motion mode of the motor. The results are depicted in Figure 13.

As can be observed in Figure 13, each excitation pulse generated a discrete angular displacement, resulting in staircase-shaped rotor-motion characteristics. The approximately horizontal sections between consecutive excitation pulses indicate that the rotor maintained its angular position after each step. This behavior confirms that the proposed motor can generate controlled incremental angular motion and retain the obtained position when the excitation signal is interrupted.

In the lower excitation range shown in Figure 13a, the mean angular resolution increased from 0.082 ± 0.015° per step at 40 V_p-p_ to 0.267 ± 0.025° per step at 60 V_p-p_. Increasing the excitation amplitude to 80 and 100 V_p-p_ resulted in mean angular displacements of 0.654 ± 0.040° and 1.219 ± 0.055° per step, respectively. After nine excitation pulses, the corresponding cumulative angular displacements were approximately 0.738°, 2.403°, 5.886°, and 10.971°. A further increase in the excitation amplitude produced larger angular steps, as shown in Figure 13b. At 120 V_p-p_, the mean angular resolution was 1.802 ± 0.075° per step. The values obtained at 140 and 160 V_p-p_ were 2.340 ± 0.090° and 2.747 ± 0.105° per step, respectively. The largest angular displacement of 3.038 ± 0.120° per step was measured at 180 V_p-p_. After nine excitation pulses, the cumulative rotor displacement reached approximately 16.218°, 21.060°, 24.723°, and 27.342° at 120, 140, 160, and 180 V_p-p_, respectively. The increase in angular displacement with excitation amplitude can be attributed to the enlargement of the elliptical trajectories of the spherical contact elements. As demonstrated by the numerical investigation, a higher excitation voltage produces larger tangential and normal displacement components at the contact interface. Consequently, a greater tangential force is transferred to the rotor during each excitation pulse, resulting in a larger angular step. The relationship between the excitation amplitude and angular resolution was nonlinear. The increase in angular displacement was particularly pronounced within the intermediate voltage range, whereas the rate of growth gradually decreased at amplitudes above approximately 140 V_p-p_. This trend is consistent with the rotational-speed characteristics and indicates the increasing influence of contact slipping, frictional losses, and other nonlinear effects at higher excitation amplitudes.

Although the absolute standard deviation increased with excitation voltage, the relative variation decreased substantially. The relative deviation was approximately 18.3% at 40 V_p-p_, but decreased to approximately 3.8–4.0% at excitation amplitudes between 140 and 180 V_p-p_. Thus, the smallest angular step was obtained at the lowest excitation voltage, whereas more stable and repeatable incremental motion was achieved at higher excitation amplitudes. All in all, the results demonstrate that the angular resolution of the motor can be controlled by adjusting the excitation-signal amplitude. Under the investigated conditions, the motor provided angular steps ranging from approximately 0.082° to 3.038°, confirming its suitability for both fine and comparatively rapid angular positioning.

Overall, the experimental results confirmed the operating principle of the proposed motor and showed good agreement with the numerical investigation. The motor reached a maximum rotational speed of 87.0 ± 2.2 RPM at 180 V_p-p_, while the angular step varied from 0.082 ± 0.015° to 3.038 ± 0.120°. These results demonstrate that both continuous rotation and incremental angular motion can be controlled by varying the excitation-signal amplitude.

During preliminary adjustment of the preload, insufficient spring compression resulted in increased slipping and unstable rotor motion, whereas excessive compression reduced the effectiveness of vibration transmission due to increased contact damping and friction. A preload of approximately 1.95 N was experimentally identified as providing stable continuous and incremental operation and was subsequently maintained constant throughout all measurements. Consequently, the rotational-speed, stall-torque, and angular-step repeatability characteristics reported in this study correspond to this fixed preload condition rather than representing a systematic dependence on preload.

The experimentally determined resonance frequency and effective electromechanical coupling coefficient of the proposed motor were 39.935 kHz and 4.47%, respectively. For comparison, Liu et al. reported measured effective electromechanical coupling coefficients ranging from 16.431% to 26.780% for four optimized stators of a 70 mm diameter hollow traveling-wave ultrasonic motor [22]. The coupling coefficient obtained in the present study is therefore considerably lower than those reported for the optimized metal–piezoelectric composite stators. However, a direct quantitative comparison should be interpreted with caution because the motors differ substantially in dimensions, stator architecture, material configuration, and operating vibration mode. In addition, Zhang et al. demonstrated that optimization of the conventional comb-tooth stator–rotor interface could increase the maximum efficiency of a traveling-wave rotary ultrasonic motor from 25% to 44.3% by reducing radial sliding [23]. In contrast, the present motor employs three discrete spherical contacts instead of a continuous toothed contact interface, thereby simplifying and localizing the mechanical interaction between the stator and rotor. Therefore, the main advantages of the proposed design are its compact coaxial architecture, direct-drive configuration, geometrically defined contact interface, and the ability to provide both continuous and incremental angular motion. Its current limitations include the comparatively low effective electromechanical coupling coefficient, the relatively high excitation voltage, and the increasing influence of contact slipping and nonlinear frictional effects at higher excitation amplitudes. Although stall torque was experimentally evaluated, complete torque–speed characteristics, mechanical output power, and efficiency were not investigated and remain subjects for future work. In addition, a systematic investigation of the influence of preload on rotational speed, stall torque, and positioning repeatability was beyond the scope of the present study and will be addressed in future work to further optimize the stator–rotor contact conditions.

Considering its compact coaxial configuration, direct-drive operation, bidirectional continuous rotation, and controllable incremental angular displacement, the proposed motor has potential for use in compact precision-positioning systems where installation volume and positioning flexibility are important. Potential applications include optical component alignment and beam-steering mechanisms, lens and filter positioning, compact gimbal and antenna-pointing systems, free-space optical communication terminals, and small robotic or mechatronic positioning stages. The ability of the rotor to maintain the obtained angular position after the excitation signal is interrupted may also be beneficial for systems requiring intermittent positioning. Nevertheless, application-specific implementation will require further investigation of output torque, load capacity, energy efficiency, thermal stability, closed-loop positioning accuracy, and long-term wear of the spherical contact interfaces.

## 5. Conclusions

The main contribution of this study is the development and experimental validation of a compact direct-drive traveling-wave piezoelectric angular motor based on a single four-electrode piezoelectric ring and three discrete spherical stator–rotor contacts, providing both continuous bidirectional rotation and controllable incremental angular positioning within the same coaxial actuator. The numerical operating frequency of 39.952 kHz closely agreed with the experimentally determined resonance frequency of 39.935 kHz, while the numerical and experimental effective electromechanical coupling coefficients were 4.36% and 4.47%, respectively. The numerical analysis confirmed that the phase-shifted excitation signals generated elliptical trajectories at the spherical contact points, providing the tangential and normal displacement components required for friction-based rotor motion. The displacement characteristics of these trajectories increased with excitation signal amplitude, which was consistent with the experimentally observed increase in rotational speed and angular step. The motor reached a maximum rotational speed of 87.0 ± 2.2 RPM at 180 V_p-p_. The stall torque increased with excitation-signal amplitude and reached approximately 8.3 N·mm and 8.4 N·mm for CW and CCW rotation, respectively, at 180 V_p-p_, confirming comparable load capability in both rotational directions. Its angular step could be controlled from 0.082 ± 0.015° to 3.038 ± 0.120° by varying the excitation signal amplitude. Although the absolute variation increased with excitation amplitude, the relative repeatability of the angular motion improved at higher excitation amplitudes. These results demonstrate the suitability of the proposed design for compact continuous-rotation and incremental angular-positioning applications.

## Figures and Tables

**Table 1:** Material characteristics used in the numerical model.

Material Properties	PZT-8	Alumina Oxide	Acrylic Plastic
Density [kg/m^3^]	7600	3900	1190
Young’s modulus [N/m^2^]	–	41.9 × 10^10^	3.20 × 10^9^
Poisson’s coefficient	–	0.25	0.35
Isotropic structural loss factor	0	–	–
Relative permittivity (ε_33_^t^/ε_0_)	1000	–	–
Elastic compliance [10^−12^ m^2^/N]	S_11_^E^ = 11.5	–	–
Elastic stiffness [N/m^2^]	C_33_^E^ = 1.468 × 10^11^	–	–
Piezoelectric constant d_33_ [10^−12^ m/V]	225	–	–
Piezoelectric constant d_31_ [10^−12^ m/V]	−97	–	–

## Data Availability

The original contributions presented in this study are included in the article. Further inquiries can be directed to the corresponding author.

## References

[1] Yasar S., Yuksel M. (2025). A Comprehensive Review of Machine Learning Applications in Laser Optics. IEEE Photonics J..

[2] Yen P., Yan M., Chen Y. (2012). Hysteresis compensation and adaptive controller design for a piezoceramic actuator system in atomic force microscopy. Asian J. Control.

[3] Mashimo T., Izuhara S. (2020). Review: Recent Advances in Micromotors. IEEE Access.

[4] Boldea I. (2020). Electric generators and motors: An overview. CES Trans. Electr. Mach. Syst..

[5] Yuan Z. (2023). Current status and prospects of actuator in robotics. Appl. Comput. Eng..

[6] Deng W., Zuo S. (2019). Electromagnetic vibration and noise of the permanent-magnet synchronous motors for electric vehicles: An overview. IEEE Trans. Transp. Electrif..

[7] Vlachou V.I., Sakkas G.K., Xintaropoulos F.P., Pechlivanidou M.S.C., Kefalas T.D., Tsili M.A., Kladas A.G. (2024). Overview on Permanent Magnet Motor Trends and Developments. Energies.

[8] Fabbro H., Le Letty R., Barillot F., Guay P., Cadiergues L. (2004). Piezoelectric actuators for active optics. (Special Publication) ESA SP.

[9] Dong S. (2012). Review on Piezoelectric, Ultrasonic, and Magnetoelectric Actuators. J. Adv. Dielectr..

[10] Niezrecki C., Brei D., Balakrishnan S., Moskalik A. (2001). Piezoelectric Actuation: State of the Art. Shock Vib. Dig..

[11] Gao X., Yang J., Wu J., Xin X., Li Z., Yuan X., Shen X., Dong S. (2020). Piezoelectric Actuators and Motors: Materials, Designs, and Applications. Adv. Mater. Technol..

[12] Koc B., Uchino K. (2016). Piezoelectric Ultrasonic Motors, no. December.

[13] Uchino K. (1998). Piezoelectric ultrasonic motors: Overview. Smart Mater. Struct..

[14] Spanner K., Koc B. (2016). Piezoelectric motors, an overview. Actuators.

[15] Le Letty R., Claeyssen F., Wendling P., Hamonic B., Bossut R. (1993). Analysis of an Ultrasonic Piezoelectric Motor Using Numerical Modeling. J. Intell. Mater. Syst. Struct..

[16] Shi S., Xiong H., Liu Y., Chen W., Liu J. (2017). A ring-type multi-DOF ultrasonic motor with four feet driving consistently. Ultrasonics.

[17] Shi S., Huang Z., Yang J., Liu Y., Chen W., Uchino K. (2018). Development of a compact ring type MDOF piezoelectric ultrasonic motor for humanoid eyeball orientation system. Sens. Actuators A Phys..

[18] Jingzhuo S., Dongmei Y. (2014). Characteristic model of travelling wave ultrasonic motor. Ultrasonics.

[19] Chen Z., Etsion I. (2020). Recent Development in Modeling of Coated Spherical Contact. Materials.

[20] Tessien R.A. (2006). Acoustic driver assembly for a spherical cavitation chamber. J. Acoust. Soc. Am..

[21] Hou P., Zhang W. (2020). The electro-mechanics of a coating/substrate system under charged spherical contact. Math. Mech. Solids.

[22] Liu J., Niu R., Zhu H., Zhao C. (2020). Improving the efficiency of a hollow ultrasonic motor by optimizing the stator’s effective electromechanical coupling coefficient. Rev. Sci. Instrum..

[23] Zhang J., Yang L., Ma C., Ren W., Zhao C., Wang F. (2019). Improving efficiency of traveling wave rotary ultrasonic motor by optimizing stator. Rev. Sci. Instrum..

